# Deciphering the *Fasciola hepatica* Glycocode and Its Involvement in Host–Parasite Interactions

**DOI:** 10.3390/biom15091235

**Published:** 2025-08-26

**Authors:** Jaclyn Swan, Timothy C. Cameron, Terry W. Spithill, Travis Beddoe

**Affiliations:** Department of Ecological Plant and Animal Science, School of Agriculture, Biomedicine and Environment, La Trobe University, Bundoora, VIC 3086, Australia; tim.cameron@latrobe.edu.au (T.C.C.); t.spithill@latrobe.edu.au (T.W.S.)

**Keywords:** glycan, glycosylation, liver fluke, immune modulation

## Abstract

The zoonotic disease fasciolosis poses a significant global threat to both humans and livestock. The causative agent of fasciolosis is *Fasciola hepatica*, which is commonly referred to as liver fluke. The emergence of drug resistance has underscored the urgent need for new therapeutic treatments against *F. hepatica*. The tegument surface of *F. hepatica* is characterized by a dynamic syncytial layer surrounded by a glycocalyx, which serves as a crucial interface in host–parasite interactions, facilitating functions such as nutrient absorption, sensory input, and defense against the host immune response. Despite its pivotal role, only recently have we delved deeper into understanding glycans at the host–parasite interface and the glycosylation of hidden antigens. These glycan antigens have shown promise for vaccine development or as targets for drug manipulation across various pathogenic species. This review aims to consolidate current knowledge on the glycosylation of *F. hepatica*, exploring glycan motifs identified through generic lectin probing and mass spectrometry. Additionally, it examines the interaction of glycoconjugates with lectins from the innate immune systems of both ruminant and human host species. An enhanced understanding of glycans’ role in *F. hepatica* biology and their critical involvement in host–parasite interactions will be instrumental in developing novel strategies to combat these parasites effectively. In the future, a more comprehensive approach may be adopted in selecting and designing potential vaccine targets, integrating insights from glycosylation studies to improve efficacy.

## 1. Introduction

The tegument of *Fasciola* spp. is surrounded by a glycocalyx, which is in direct contact with the host immune system; however, it is only recently that the role of this dynamic layer and other glycosylated tissues have begun to be characterized and studied with regards to host–parasite interactions. Firstly, fasciolosis is a globally distributed disease that causes substantial economic losses in livestock, with the causative agents being *Fasciola hepatica* and *Fasciola gigantica*. *Fasciola hepatica* is primarily distributed in temperate regions worldwide, although it also prevails in certain countries with tropical climates. On the other hand, *F. gigantica*, which is less extensively studied, is typically found in tropical regions. The distribution of these two parasites is thought to be separated in part due to differences in the distribution of the semi-aquatic snail species that are the intermediate host [1]. However, it has recently been shown that this perceived distribution is blurred due to hybridization events occurring between these two species [2,3]. *Fasciola* spp. infect a great number of different mammals, with the most economically significant being sheep and cattle. Alarmingly, humans can also become infected, and now the WHO recognizes fasciolosis as an emerging neglected tropical disease, with an estimate of 180 million people at risk of infection [4].

Humans and livestock become infected following the ingestion of vegetation-encysted metacercariae, which are likely located in close proximity to a water source inhabited by the intermediate snail host. Following ingestion, the metacercariae excysts in the small intestine progress into newly excysted juveniles (NEJs) and burrow through the gut wall and peritoneal cavity to enter the liver. Approximately 6–8 weeks following ingestion, the NEJs continue to burrow through the liver tissue. This causes a significant amount of liver damage, calcification, and anemia. Subsequently, the immature fluke migrates to the bile duct where it matures into a sexually reproductive adult, leading to the secretion of eggs into the feces. These intra-mammalian life stages are responsible for substantial economic losses amounting to billions of dollars annually to the global livestock industry [5]. These estimates are derived from factors such as diminished growth rates, reduced milk and wool yields, and decreased fertility observed in infected animals [6,7].

Unfortunately, drug resistance to a number of anthelmintics is now a common global issue, with cases of humans infected with these drug-resistant strains [8]. Alternative treatment methods are urgently needed. A vaccine to prevent infection and associated economic losses would be very beneficial; however, the host–parasite interactions are very complex and are proving difficult to overcome in the development of an efficacious vaccine [9]. Increasing our knowledge of the host–fluke interface would drastically improve the chances of designing a successful vaccine. In order to produce an effective vaccine, a deep understanding of the immunology associated with *F. hepatica* infection is needed. Following the ingestion of *F. hepatica* metacercariae, a strong type 2 immune response is stimulated that is generally characterized by anti-inflammatory cytokines such as IL-10 and IL-4 and a decreased expression of MHC class II molecules and other surface molecules such as CD86 by immune cells, triggering the induction of alternatively activated macrophages, anergic T cells, and decreased IFN_ϒ_ [10,11,12,13].

Therefore, understanding the molecular mechanisms of infection and immunomodulation would aid in the development of novel treatment and prevention options. Interestingly, carbohydrate receptors on the cells of both the mammalian and snail host are thought to be involved in infection by *F. hepatica*. For example, when NEJs were pre-incubated with the mannose binding lectins concanavalin A (ConA) and *Galanthus nivalis* lectin (GNL), migration through intestinal tissue was inhibited [14]. Similarly, when naïve *Pseudosuccinea columella* (a common intermediate host of *F. hepatica*) hemocytes were pre-incubated with the monosaccharides mannose or fucose, this prevented these snail immune cells from encapsulating *F. hepatica* sporocysts [15]. Evidently, parasite glycans are an important component of the host–parasite interaction in both the definitive and intermediate host.

The role that *F. hepatica* glycans play in primary host interactions are also starting to come to light. It has previously been shown that parasitic glycans, including those of *F. hepatica,* have been shown to play a role in inducing a Th2-like immune response [16]. Glycans present in *F. hepatica* excretory/secretory (FhES) products have been demonstrated in vitro to play a role in the alternative activation of peritoneal macrophages from BALB/c mice. Activation was characterized by the increased expression of arginase 1, transforming growth factor β (TGF-β), and IL-10 [17]. It was demonstrated that these effects could be inhibited by pre-incubating the cells with sugars such as mannan and laminarin or antibodies such as anti-mannose receptor or anti-dectin-1 receptor, demonstrating that a glycan–protein interaction can play a role in stimulating an anti-inflammatory immune response. The host interacts with parasite glycans through a number of different protein receptors known as lectins. These lectins can be both soluble or membrane bound and are present within many different innate immune cells, including macrophages and dendritic cells [16,17,18].

Glycosylation of vaccine antigens is rarely given the consideration it deserves; alternatively, the only consideration glycans are often given is to just remove the non-native N-linked glycans of recombinant antigens using endoglycosidases. This can have a considerable effect on the protein’s characteristics and function such as altered conformation, solubility, or ligand interaction [19,20]. In view of the difficulties of producing a commercially viable liver fluke vaccine, the expectation of 100% protection is unlikely to be feasible; however, a reduction in egg production and parasite viability will aid in management and significantly reduce production losses due to fasciolosis [9]. A partially protective vaccine will also reduce the reliance on anthelmintics, aiding in slowing the development of drug resistance and reducing residues in meat and milk [9,21]. The majority of vaccines to date have focused on protein antigens, both native and recombinant. However, it is evident that native vaccines may have greater efficacy than their recombinant orthologs and potentially greater reproducibility [9,22]. This strategy is reflected by the success of the BarberVax^®^ vaccine used to combat the gastrointestinal nematode *Haemonchus contortus* [23]. It is a crude native glycoprotein extract [23,24], and to date, the efficacy of the native glycoproteins cannot be replicated in recombinant systems [25,26]. This is likely attributed to several factors, with the failure to accurately replicate native glycosylation possibly playing a significant role [25]. Some exciting advances are being made in this area by glycoengineering insect cell systems that produce highly fucosylated nematode-like glycoproteins [27], with promising results.

Although a significant amount of data has been generated with regards to some aspects of *Fasciola* glycans, there is still much to be discovered. Further analysis of *Fasciola* glycans will deepen our knowledge of host–parasite interactions and likely aid in the development of new drugs and vaccines against *Fasciola*. This review will collate the current research that has been conducted with regards to *Fasciola* glycans and highlight the key areas where future glycan research should be focused to deepen our understanding of host–fluke interactions.

## 2. Glycan Machinery: Glycosyltransferases and Glycosidases

**Figure 1:**
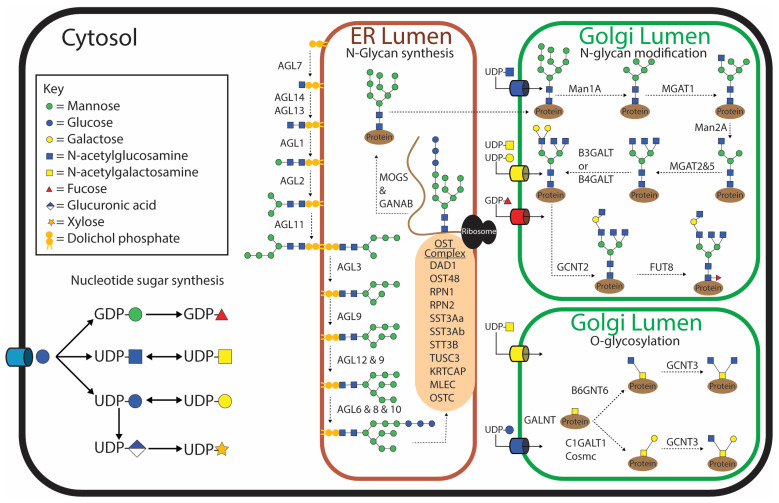
Depiction of the current understanding of glycosylation pathways within *Fasciola hepatica* based on in silico analysis identifying orthologous genes that encode glycosylating enzymes of humans identified within the *F. hepatica* genome [28].

To date, the glycosylation pathways have not been well characterized for any parasitic helminth. However, knowing which enzymes involved in glycosylation are present in a parasite and their function would allow for a narrower search field when performing glycomic analyses and potentially provide novel candidates for drug and vaccine targets. Currently, the most complete repertoire of known enzymes involved in glycosylation for any parasitic helminth is collated for *F. hepatica* [28] (Figure 1). By employing an in silico analysis to scrutinize the *F. hepatica* genome against 190 human genes associated with glycosylation, 153 orthologous *F. hepatica* glycosylating genes were identified. Only 87 were labeled as true orthologs, and 31 of these had multiple paralogs, thought to be due to gene duplication events, which is a common occurrence for *F. hepatica* and other trematodes [28,29]. However, a sequence does not always infer function, which is particularly true of glycosyltransferases, as two sequences with high sequence similarity can differ in catalytic activity [30], but currently, this is the only information available for helminths. The data suggest that *F. hepatica* appears to have the genes required for general glycosylation such as sugar transporters needed to transport monosaccharides into the cell, nucleotide sugar synthesis enzymes necessary to activate the individual monosaccharides, and nucleotide sugar transporters needed to transport the activated monosaccharides from the cytosol into the endoplasmic reticulum (ER) or Golgi (Figure 1). The exception to this is that the four mammalian enzymes involved in CMP-N-acetylneuraminic acid (CMP-Neu5Ac) synthesis do not have similarity to any *F. hepatica* genes, although a gene with similarity to a CMP-Neu5Ac transporter was identified [28]. The current prevailing opinion is that *F. hepatica* or any other helminths do not synthesize CMP-Neu5Ac [31]. It would be interesting to explore if *F. hepatica* can obtain sialic acid from the digested cells of its host; previously, this has only been demonstrated for the protozoan parasite *Trypanosoma cruzi* [32]. This would explain the presence of the CMP-Neu5Ac transporter despite the absence of any enzymes able to synthesize CMP-Neu5Ac in *T. cruzi*, although enzymes to activate the monosaccharide are also lacking.

### 2.1. N-Glycan Synthesis

Generally, the synthesis of N-glycans begins with the production of the lipid-linked oligosaccharide precursor on the cytosolic side of the ER membrane by the addition of two N-acetylglucosamines (GlcNAcs) and five mannose (Man) molecules to a dolichol-phosphate [19]. The growing polysaccharide is then flipped inside the ER, and an additional four mannose and three glucose (Glc) molecules are added. This process is catalyzed by asparagine-linked glycosylation (ALG) glycosyltransferases. N-Glycosylation of secreted and membrane proteins is then initiated in the ER by the transfer of the lipid-linked oligosaccharide precursor onto the asparagine within the consensus sequence Asn-X-Ser/Thr (where ‘x’ is any amino acid except proline) of the nascent polypeptide. This ‘en bloc’ transfer is catalyzed by the oligosaccharyltransferase (OST) complex. Although this is the generic pathway shared by most eukaryotes, variations in this process have been observed in some protozoan parasites, although these variations do not seem apparent in *F. hepatica* [33]. McVeigh, et al. [28] identified homologs of all the ALG genes and nine OST genes, including the five that are essential for the survival of yeast [34]. This implies that *F. hepatica* produces the generic lipid-linked Glc_3_Man_9_GlcNAc_2_ structure prior to en bloc transfer onto the nascent polypeptide. Genes homologous to all the mammalian glycosidases involved in trimming down the Glc_3_Man_9_GlcNAc_2_ to Man_3_GlcNAc_2_ are present in the *F. hepatica* genome [28] (Figure 1). The potential for hybrid structures exists due to the presence of a homolog of MGAT1 (GlcNAc-T1), which adds a β2-GlcNAc to the α3-mannose on the structure prior to the removal of the final two mannose molecules on the α6-mannose arm [28]. No gene homolog for the human MGAT4 gene was identified in *F. hepatica*, suggesting that this arm cannot be branched by a β1,4-GlcNAc. Similarly, a homolog to the gene that adds a bisecting GlcNAc to the core mannose could not be identified (MGAT3) [28]. However the α6-mannose arm can be modified by one, or by a combination, of the β2-GlcNAc (MGAT2) or β6-GlcNAc (MGAT5) gene products; interestingly, five paralogs of MGAT5 were identified [28]. These results suggest that triantennary N-glycans may be possible but not tetraantennary N-glycans. It is thought that the terminal GlcNAc can be extended with either β1,3-Gal or β1,4-Gal molecules by either B3GalT or B4GalT, respectively, giving rise to both type 1 and type 2 N-acetyllactosamine (LacNAc) motifs, with many paralogs of each. As humans do not possess the enzyme to transfer galactose in a β1,6 linkage conformation, this enzyme was not used as a search query. However, this motif has appeared on glycolipids of *F. hepatica* [35]; therefore, it would be interesting to also identify this enzyme within the *F. hepatica* genome. The antennal galactose can potentially be extended further by the addition of a β1,6-GlcNAc by GCNT2.

It appears that these N-glycans can have a core α1,6 fucose modification to the asparagine-linked GlcNAc due to two paralogs of the mammalian FUT8 being identified in *F. hepatica*. What was very interesting is that no similarity to an α1,3 fucosyltransferase could be identified in the *F. hepatica* genome. This is surprising as this motif is very well characterized in *Schistosoma mansoni*, a closely related trematode, and has been shown to be very dominant and highly antigenic. Similarly, a β1,2 xylosyltransferease known in *S. mansoni* has not been found in *F. hepatica*, but as this is not present in humans, it was therefore not used as a search query. The lack of a core α1,3 fucose modification in *F. hepatica* has been supported by the PNGase A treatment of tegument extracts from adults and NEJs, where PNGase A is an exoglycosidase that preferentially removes N-glycans that are modified with a core α1,3 fucose [36,37].

### 2.2. O-Glycan Synthesis

O-Glycosylation is initiated by the transfer of N-acetylgalactosamine (GalNAc) to a serine or threonine residue [38]. This is commonly referred to as a Tn antigen and is catalyzed by GALNT (ppGaNTase) (Figure 1). Humans have 18 orthologs of GALNT, whereas *F. hepatica* appears to only have six, but of these, they have many paralogs [28]. GALNT is one of the very first glycosyltransferases to have activity demonstrated in *F. hepatica* and was demonstrated to have greater preference for human MUC2 and MUC6 and a mucin peptide from *T. cruzi* and *T. brucei* compared to human MUC1, MUC2, and MUC5b or a synthetic *S. mansoni* glycopeptide [39]. In a normal O-glycosylation pathway, the Tn antigen can be extended into either a core 1 or core 3 structure. Thirteen different paralogs of C1GAlT1 were identified in *F. hepatica* including the compulsory chaperone Cosmc, which is needed for transport of this enzyme from the ER to the Golgi to catalyze the formation of the core 1 structure [40]. Only one ortholog of B3GNT6 was identified to create a core 3-type O-glycan. Both core 1 and core 3 can be modified by GCNT3 to form core 2 and core 4 structures, respectively [28], and *F. hepatica* has six paralogs of this enzyme. These account for the commonly identified O-glycans; however, unusual O-glycans have also been identified in other parasites, and therefore, the potential for non-mammalian modifications exists in *F. hepatica*, which has not been explored.

RNA-seq data suggest that glycosylation genes are differentially regulated during the different intra-mammalian and metacercariae liver fluke life stages [28,29,41]. The in silico dataset has demonstrated the plausibility of the dominant oligomannose N-glycans and has suggested that *F. hepatica* glycans are less complex than humans [28]. However, in the future, the inferred function of these homologous glycosylating gene products should be verified by biochemical assays and in-depth glycomic analysis in order to validate the in silico analyses and have greater confidence in the identification of glycans being expressed by *F. hepatica*.

## 3. Characterization of Glycans Using Generic Lectins

The glycocalyx of *F. hepatica* was first described by Threadgold [42] and was thought to be anionic, speculated to be due to the presence of sialic acid and sulphated glycans observed by staining with cationic dyes and periodic acid-Schiff techniques. Since then, almost every life stage of *F. hepatica* has been probed with at least one generic lectin (Table 1). This has given some insight into which monosaccharides are likely to be present and, in some cases, also where they can be expected. Different techniques have been used such as lectin arrays, lectin blots, or lectin histochemical analyses. Due to this and the nature of lectin affinity, some studies contradict one another, although in most cases, and especially for the more widely studied life stage of the adult, this is less frequent.

For the more thoroughly studied adult life stage, the most abundant and widely dispersed lectin binding is seen by mannose binding lectins such as ConA, *Lens culinaris* agglutinin (LCA), *Pisum sativum* agglutinin (PSA), and GNL, which bind to all features on the outer surface of the fluke and internal tissues [43,44]. Lectins that interact with terminal GlcNAc and GalNAc show a very similar distribution; however, the staining intensities vary much more compared to that of mannose binding lectins [43,44]. Interestingly, both terminal fucose and terminal galactose binding lectins appear to be more isolated to the spines/spinelets and surface coat of the tegument but did not show binding to the ventral or oral suckers [44]. In contrast, lectin binding suggests that glycoconjugates with α2-3 sialic acid are isolated to the spines/spinelets only [44], which is surprising as enzymes to make this glycan modification have not been identified. The localization of different glycan motifs demonstrates the differential glycosylation of *F. hepatica* surface structures, suggesting that specific glycan motifs could partake in distinct functions.

**Table 1:** Glycan motifs in *Fasciola hepatica* identified by generic lectins.

Life Stage	Glycan Motif	Lectin	Reference
Egg	(Fuc α1,2)Gal β1,4 Glc	BaSII	[45]
Miracidium	Oligomannose	ConA, LCA	[46]
	GlcNAc	WGA, LEL	[46]
	(Fuc α1,2)Gal β1,4 Glc	BaSII	[45]
	No binding	SBA, HPA, UEA-1	[46]
Sporocyst	Oligomannose	ConA, LCA	[47]
	No binding	WGA, SBA, UEA-I	[47]
Rediae	GalNAc/Gal	SBA	[48]
	No binding	ConA, LCA, WGA, UEA-I	[46]
Cercariae	No Data		
Metacercariae	(Fuc α1,2)Gal β1,4 Glc	BaSII	[45]
Newly Excysted Juvenile	Oligomannose	ConA, GNL, LCA, PSA	[37,49,50]
	GlcNAc	GLS-II, WGA, S-WGA	[37,49,50]
	GalNAc/Gal	GSL-I, SBA, DBA, VVL, SJA	[37,50]
	β-linked Gal	PNA, ECL, Jacalin	[37,50]
	Fucose	AAL, UEA-I	[37]
	Complex	PHA-L, PHA-E,	[37]
Adult	Oligomannose	NPA, HHA, ConA, PSA, LCA, GNL	[16,43,44,51]
	GlcNAc	GSL-II, WGA, s-WGA, STL	[16,43,44,51]
	Chitobiose or N-Acetyllactosamine	LEL, DSA	[43,44]
	GalNAc/Gal	GSL-I, SBA, DBA, VVL, SJA, SNA-II, WFA	[16,43,44,52]
	β-linked Gal	RCA-I, PNA, PHA-E, Jacalin, ECL, SJA	[16,43,44]
	Terminal α-linked Gal	GSL-1-B4, MPA, VRA, MOA	[44]
	Fucose	AAL, LTA, UAE-1	[43,44,52,53]
	(Fuc α1,2)Gal β1,4 Glc	BaSII	[45]
	Complex glycans	PHA-L, PHA-E, CPA	[44]
	Sialic Acid	SNA-I, MAL-I, MAL-II	[44]

### Extracellular Vesicle (EV) Glycan Topology Determined with Lectin Microarrays

Another mechanism by which *F. hepatica* modulates the host immune system is through the release of extracellular vesicles (EVs), which encapsulate proteins, lipids, carbohydrates, and RNA within a lipid bilayer. This packaging protects the cargo from rapid degradation in the host’s extracellular environment and enables it to interact with the host at sites distant from the parasite itself [54,55]. The glycoproteins and glycolipids embedded in the lipid bilayer of the EV are thought to play a role in docking to the host immune cells and internalization. Live adult flukes were collected and incubated in media for 5 hrs, and the EVs were collected by either sequential centrifugation or the gravity flow method [56]. To determine what glycan motifs are displayed on the outside of the *F. hepatica* EVs, they were fluorescently labeled and passed over a lectin microarray consisting of 48 lectins. Similar to the adult fluke tegument, strong binding was observed for lectins that recognize terminal mannose residues, such as NPA, GNL, HHA, and PSA, as well as those targeting N-glycans, complex-type glycans, and terminal galactose, GlcNAc, and GalNAc structures [56,57]. Each of the EV subpopulations (15 k and 120 k), as well as the EVs collected by gravity flow, showed very similar lectin recognition ability, suggesting that the glycans of each are similar to, but different from, that of the tegument. Surprisingly, a similar lectin array has been used to determine the glycan motifs presented by *S. mansoni* EVs demonstrating similar, if not slightly less, diverse glycosylation patterns [58]. *Schistosoma mansoni* EVs showed more binding to SNA-I (a lectin that specifically recognizes α2-6 sialic acid) than *F. hepatica*, which is not believed to be synthesized by either of the trematode species, although *F. hepatica* does possess a gene with homology to a CMP-sialic acid transporter. Sialic acid is potentially incorporated by the parasite via pinocytosis and a lysosomal transporter, similar to how humans can incorporate the non-human sialic acid N-glycolylneuraminic acid (Neu5Gc) from dietary sources [59,60,61]. The glycans on the surface of the EVs are resistant to exo- and endogylcosidases suggesting that the glycans could be highly modified [57]. Treatment of the EV surface with PNGase F did slightly alter the lectin recognition and significantly reduced the uptake of the EVs by rat macrophages, demonstrating that N-glycans play an essential role in the internalization of *F. hepatica* EVs by host immune cells [57].

## 4. Mass Spectrometry (MS) Characterization of Fasciola Hepatica Glycans

**Figure 2:**
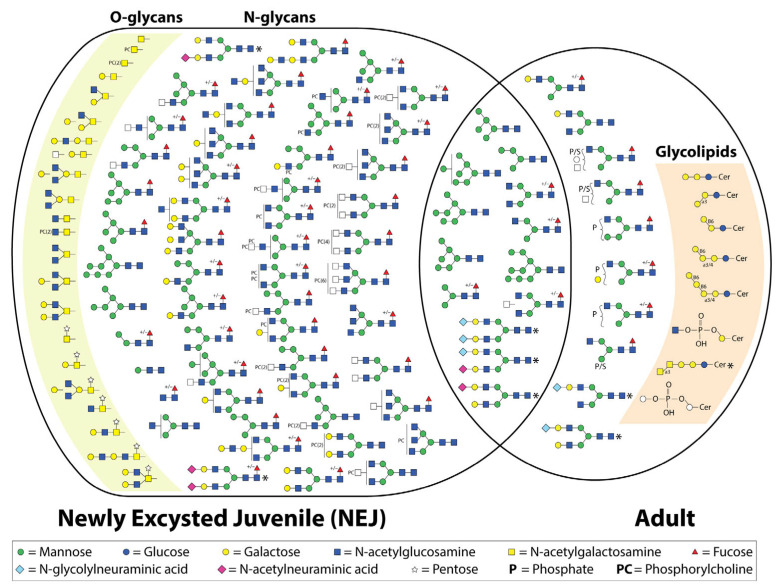
Depiction of the structures currently identified by mass spectrometry in newly excysted juvenile and adult *Fasciola hepatica* [18,36,37,50,64]. +/− indicates whether the structure was detected both with and without α1-6-linked core fucose. Structures identified that are thought to be the result of host contamination are marked with an asterisk.

Although generic lectins have informed us about potential glycan structures present within different lysate preparations and life stages of *F. hepatica*, mass spectrometry (MS) analysis is needed for a deeper understanding of glycan composition and structure (Figure 2). For many glycan motifs, lectins with enough specificity or affinity just do not exist. To date, most MS studies of *F. hepatica* have focused on N-linked glycans [18,36,37,44]. Only recently have several O-linked glycans been characterized, alongside the identification of both N- and O-linked glycopeptides [50]. Additionally, a small number of glycolipids have been characterized in considerable detail [35,62,63]. So far, only the NEJ and adult life stages have been examined by MS. Consequently, no detailed glycan information exists for the free-living or intra-molluscan life stages, other than what has been indicated by lectin staining.

### 4.1. N-Linked Glycans

To date, all studies, regardless of the life stage examined, have reported a dominance of oligomannose and paucimannose structures, with the addition of a few hybrid or complex structures with terminal GlcNAc [18] or galactose [36]. Garcia-Campos, et al. [37] investigated the N-linked glycans of a tegument extract of newly excysted juvenile flukes. The extract was treated with PNGase F and labeled with 2-aminobenzoic acid (2-AA), followed by MALDI-TOF MS in negative ion mode. This was followed by treatment with a number of exoglycosidases to integrate the presumed glycan composition. Sixteen N-glycans were identified (Figure 2), the majority of which were either oligomannose or paucimannose structures [37].

The same procedure was used by Ravidà, et al. [36], who interrogated the N-linked glycans of tegument extracts from adult *F. hepatica*. An alternate method was employed by Rodríguez, et al. [18], who used a 2D LC MS/MS to identify PNGase F-released N-glycans of adult whole worm extracts (FhWWEs) collected from sheep. However, one limitation is that exoglycosidases were not utilized. In both cases, a dominance of oligomannose and paucimannose structures was reported, with the addition of a few hybrid and complex structures (Figure 2). The former study was the first to detect anionic structures by mass spectrometry. To determine if these structures were sulphated or phosphorylated, high-resolution MALDI FT-ICR MS was used, revealing that these anionic structures were phosphorylated. MALDI-TOF MS/MS was employed to identify the phosphorylated monosaccharides of four selected N-glycan species. The analysis revealed phosphorylation of both a hexose and an N-acetylhexosamine (HexNAc), presumed to be mannose and GlcNAc, respectively, based on the biosynthetic pathway. Additionally, the phosphorylated GlcNAc could not be digested by β-hexosaminidase [36]. This is not the first time that anionic glycans have been reported in *F. hepatica*; Threadgold [42] had demonstrated their presence by staining with cationic dyes, while Ravidà, et al. [36] also revealed glycans containing terminal galactose by digestion with β-galactosidase.

More recently the N-glycome (released by PNGase F from somatic and secreted extracts) of *F. hepatica* NEJs has been re-investigated via LC-ESI-MS/MS [50]. Both released N-glycans (PNGase F treated) and intact glycopeptides were analyzed. This has provided a far more complete view of the variety of N-glycans presented by NEJs. In total, 53 N-glycan compositions were detected in both the N-glycan and glycopeptide analysis [50] (Figure 2). In conjunction with previous studies, the majority of the N-glycans observed were oligomannose and paucimannose/truncated species. Similarly, terminal galactose was detected as well as poly LacNAc extensions. Surprisingly, no phosphorylated N-glycans were detected, which had been seen previously in adult flukes [36]. However, a number of more unusual N-glycan species were also detected, such as phosphocholine (PC) modifications on HexNAc. Even multiple PCs were detected on a single HexNAc [50]. The identity of the modified underlying HexNAc was not concluded. The same study demonstrated that the PC motifs are abundantly displayed over the surface of the tegument and therefore presumed to be involved in host–parasite interactions [50]. This is not the first time PC has been proposed to modify *F. hepatica* glycans; it was first detected in immature and adult extracts using an ELISA [65]. The role this modification could be playing is open to speculation; however, the ES-62 antigen, a glycoprotein containing PC, of the filarial nematode *Acanthocheilonema viteae* has a remarkable efficacy in inducing an anti-inflammatory immune response [66,67]. Additionally, it is thought that this PC modification could be preventing activation of the lectin pathway of the complement system [50,65]. The authors suggest that targeting this pathway could provide novel treatments [50].

All of these studies have identified N-glycans with terminal sialic acid, including both Neu5Ac and Neu5Gc. While sialic acid has been previously detected by several lectins, it remains challenging to definitively rule out the potential for host contamination of these structures [36,37,44,50]. The incorporation of host-derived sialic acid warrants further investigation. The origins of non-sialyated mammalian-type glycans when the adult flukes are derived from cattle and sheep [18,36,44] are more difficult to conclude. All studies have only detected core α1-6 fucose, which correlates with the lack of a homologous α1-3 fucosyltransferase. Regardless, it is very interesting that *S. mansoni* has both α1-3 and α1-6 core fucose and commonly has branching and terminal α1-3 fucose [68], which *F. hepatica* appears to lack.

Previous studies of the N-glycans of *F. hepatica* lacked separation by liquid chromatography, which prevented the identification of isobaric compositions, isomeric structures, or structures of very similar mass [36,37]. The consequence of this was observed when using the more sensitive high-resolution MALDI-FT-ICR MS, which revealed a number of closely related glycan masses that previously were over-dominated by more abundant structures due to a lack of resolution of structures with similar masses [36]. However, it is now evident from more thorough analyses that the N-glycome of *F. hepatica* is far more complex than initially believed, containing numerous non-mammalian N-glycan structures. This complexity suggests that such glycans may be overlooked by comparing to a human-type glycome or the in silico glycosyltransferase analyses discussed earlier. It will be intriguing to explore how the N-glycome of *F. hepatica* varies across its different life stages in future studies.

### 4.2. O-Linked Glycans

Very few O-glycans of parasites have been identified. Initially, the only O-glycans identified in *F. hepatica* were the Tn antigen and sialyl-Tn antigen, which were detected by using monoclonal antibodies in which western blots demonstrated multiple proteins were post-translationally modified with these O-glycans [39]. However, immunohistochemistry showed sialyl-Tn staining in the gut of the adult fluke; therefore, it could potentially be host derived. Recently De Marco Verissimo, et al. [50] used β-elimination and LC-ESI-MS/MS to characterize the O-glycans of the ES and somatic extract of NEJs (Figure 2). O-Glycans with type 1, 2, 3, and 4 core structures were identified as well as the previously detected T and Tn antigen O-glycans. Similar to the N-glycans, PC modifications were found to decorate the HexNAc, which is believed to be GlcNAc. Most surprising was the identification of a pentose, assumed to be xylose, on the reducing GalNac. Xylose has previously been identified in a β1-2 linkage to the mannose proximal to the chitobiose core of N-glycans derived from *S. mansoni* and various plant and insect species; this epitope is quite antigenic in host species [69]. However, the pentose identified on O-glycans of *F. hepatica* could not be removed with a β1-2 xylosidase, suggesting an alternative linkage [50]. The gene with homology to the transcript to create UDP-xylose has been identified in the *F. hepatica* genome [28], yet the glycosyltransferase is completely unknown. The location of the pentose monosaccharide was confirmed with MS/MS analysis and demonstrated that this was not a glycosaminoglycan core structure [50]. The addition of pentose to O-glycans appears to be quite common for *F. hepatica* NEJs, with this modification present on ~43% of the of the total O-glycan structures identified. The antigenicity of this glycan motif will be interesting to investigate in the future.

### 4.3. Glycopeptide Analysis

The first glycopeptide study was conducted by Garcia-Campos, et al. [37], who demonstrated that cathepsin B1 (FhCatB1) and cathepsin L3 (FhCatL3_4) are modified with paucimannose structures in NEJs. Electron capture dissociation (ECD) spectra suggested possible core fucosylation of the glycan; however, electron transfer dissociation (EDT) fragmentation supporting glycosylation of this particular peptide could not be obtained [37]. At the time, the lack of a dedicated *F. hepatica* glycan library made global glycopeptide analysis either labor-intensive or poorly representative when relying on mammalian or plant libraries.

To overcome the lack of an *F. hepatica* N-glycan library, a more recent study combined a released N-glycan and O-glycan dataset with intact glycopeptide analysis [50]. Surprisingly, greater N-glycan coverage was found in the glycopeptide analysis compared to the released N-glycan dataset. Nevertheless, 365 unique glycopeptides were detected, making up 123 glycoproteins, some with more than one occupied glycosylation site with an immense amount of micro-heterogeneity at each site [50]. However, the variation at N-glycosylation sites was greater than at O-glycosylation sites. In total, 112 glycoproteins were detected with N-glycans, 6 glycoproteins with O-glycans, and 6 glycoproteins had both N- and O-glycosylation sites occupied. The dominance of N-glycopeptides may reflect a bias introduced by hydrophilic interaction chromatography (HILIC), which was used to enrich for glycopeptides [50]. As with any enrichment method, HILIC can introduce selectivity, in this case, favoring large glycans or peptides with multiple occupied glycosylation sites. This can lead to an underrepresentation of paucimannose N-glycopeptides and all small O-glycopeptides [70]. An alternative approach is boronic acid-based enrichment, though this has the drawback of being a relatively weak interaction, which can result in loss of low abundant glycopeptides [70]. Regardless, FhCatL3_4 and FhCatB1 were both detected in this study as well, with more glycoforms on these proteins observed this time [37,50]. Interestingly the somatic extract was found to have less processed N-glycans than those observed in the secreted/excreted extract.

Many of the glycoproteins detected had been included in previous vaccine trials. In the future, if the micro-heterogeneity of glycopeptides could be distinguished for potential native glycoprotein vaccine candidates, and if the antigenicity of the dominant glycoforms could be assessed, a thoroughly informed decision could be generated to assess the feasibility of either glycoengineering the candidate or using a de-glycosylated/modified form.

### 4.4. Glycolipid Mass Spectrometry

More detailed analyses of several *F. hepatica* glycolipids have been conducted. This has demonstrated the presence of both neutral and acidic glycolipids, some of which have been shown to be antigenic, with biomarker potential for serodiagnosis [35,62,63]. Initially, it was presumed that the glycolipids of *F. hepatica* would resemble those previously identified in *S. mansoni*. However, contrary to expectations, *F. hepatica* does not seem to possess fucosylated lipid glycoconjugates despite the similarity in lipid structures [63]. Three mammalian-type lipid glycoconjugates have been identified, including globotriaosylceramides (Galα1-4Galβ1-4Glc1-1 ceramide), isoglobotriaosylceramides (Galα1-3Galβ1-4Glc1-1 ceramide), and the Forssman antigen (GalNAcα1-3GalNAcβ1-4/3Galα1-4/3Galβ1-4Glc1-1 ceramide) (Figure 2). However, unlike the previous two glycoconjugates that were found to be attached to parasite-like phytosphingosine and α-hydroxylated fatty acids, the Forssman antigen was found to be attached to a mammalian-type lipid, making the source of this glycolipid questionable [35,63]. This was confirmed by analyzing host (sheep) lipids, and specific staining demonstrated that they were isolated in the gut of *F. hepatica* [35]. Interestingly, globo-series glycosphingolipids (GSLs) are present on host lymphocytes; it has been speculated that *F. hepatica* is also expressing this antigen (CD77/Gb3) as either a way of appearing ‘host-like’ or alternatively stimulating an immune response that will also target host lymphocytes [63].

Non-mammalian glycolipids have also been identified, including Galβ1-6Galβ1-4Glc1-1 ceramide, Galβ1-6Galα1-3/4Galβ1-4Glc1-1 ceramide, and Galβ1-6Galβ1-6Galα1-3/4Galβ1-4Glc1-1 ceramide (Figure 1) [35,63]. The terminal β6-linked galactose is a motif also identified in tapeworms and is thought to potentially be responsible for the antigenic cross-reactivity between *F. hepatica* and *Echinococcus granulosus*, as well as *Taenia crassiceps* [35]. Lastly, an acidic glycolipid GlcNAcα1-HPO3-6Gal1-1 ceramide has been identified, which was demonstrated to be highly reactive with sera from both *F. hepatica*-infected rabbits and humans [62]. Each of these studies has been conducted on adult *F. hepatica* implementing all or a combination of techniques including fractionation, MALDI-TOF, offline ESI-MS, enzymatic cleavage, NMR, and immunohistochemistry to provide in-depth analysis [35,62,63].

More recently, mass spectrometry imaging (MSI) was performed on cross sections of adult flukes and migrating immature flukes within rat liver tissue [64]. In addition to detecting HexNAc-HPO_3_-Hex-ceramide [62], Hex-HPO_3_-Hex ceramide was also identified in both adult and immature flukes. These phosphorylated glycolipids may be excreted during the migratory phase, as they were observed in association with hepatic tissue lesions. This same study also identified a number of other glycosylated ceramides (HexNAc_(0–7)_Hex_(1–4)_ ceramide) with differing spatial distributions throughout the fluke, showing some quite specific to the tegument [64] and highlighting the benefit of spatial mass spectrometry.

### 4.5. Concluding Remarks on the Currently Identified Glycan Structures

In recent years, the knowledge of the glycosylation of *F. hepatica* has come a long way from the staining of the mucopolysaccharide layer and initial lectin immunohistochemistry. The unusual (non-mammalian) modifications of the glycan structures mentioned above offer potential new targets for therapeutics and an opportunity to learn about the function of such modifications. Despite this, there is so much more to discover. For example, whole glycan classes are missing from the *F. hepatica* literature (and most parasites, for that case), such as glycosaminoglycans (GAGs) and glycosylphosphatidylinositol (GPI). GAGs are repeating disaccharide units attached to a proteoglycan by a linkage region tetrasaccharide [71]. Chondroitin repeating units have been detected in *C. elegans;* however, they are generally not sulphated, which is uncommon in mammalian species. Previously GAG-like O-glycans have been detected in the nematode *Oesophagostomum dentatum* in pigs [72]. The presence and diversity of these glycan classes in *F. hepatica* will be interesting to investigate in the future. Despite its utility, LC-MS glycomics faces key limitations, including incomplete isomer separation, challenges in linkage analysis (requiring cross-ring fragmentation or exoglycosidase digestion), and difficulties in quantification due to variable ionization efficiencies or the use of diverse fluorescent tags, while reliance on manual annotation remains time-consuming and prone to bias. Nevertheless, the increasing sensitivity of today’s MS instruments and open mindedness to non-mammalian glycan structures will allow a more comprehensive investigation of this area.

## 5. Host–Parasite Immune Interactions

**Figure 3:**
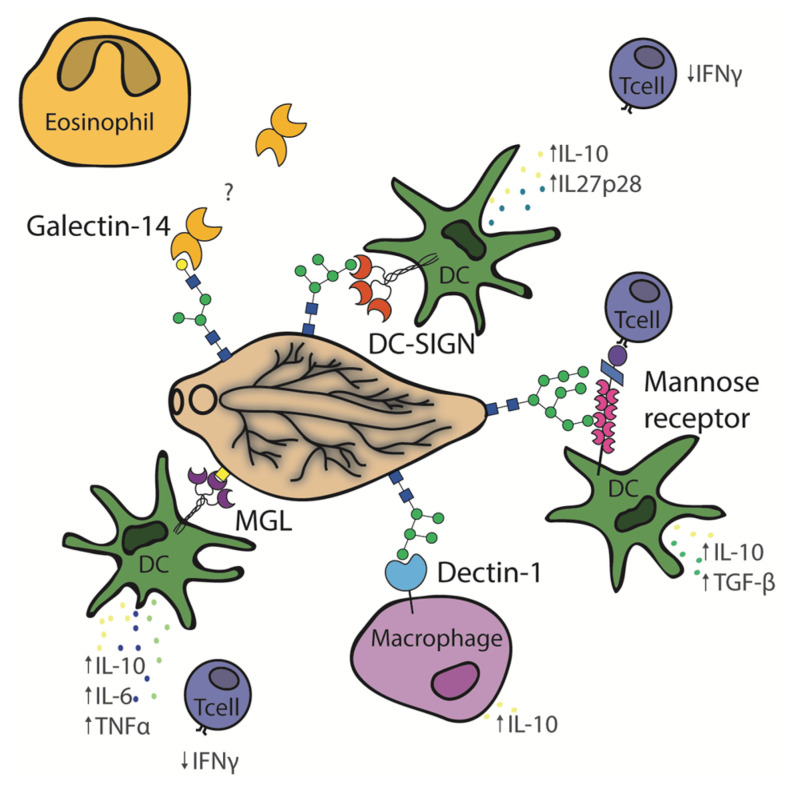
Schematic depiction of *Fasciola hepatica* glycan interactions with the immune lectins, MGL, DC-SIGN, dectin-1, mannose receptor, and galectin-14.

Mammalian innate immunity carries a substantial burden for the defense function against pathogens, and therefore, it has evolved a range of receptors to detect these pathogens and counteract them. These receptors are termed pattern recognition receptors (PRRs), which bind microbial and parasite surface molecules such as glycans. The parasite glycans interact with the immune system through a number of different host lectins present on the surface of a range of immune surveillance cells. For many lectins, the glycans that they specifically interact with are currently unknown, and the signaling cascade triggered by this interaction is also indistinct. The current knowledge of host immune lectin interactions with *F. hepatica* is outlined (Figure 3).

### 5.1. Dectin-1

Dectin-1 is a C-type lectin that has been shown to act as a PRR and interact with fungal β-glucan and has been reported to also interact with *F. hepatica* [73,74]. Dectin-1 is expressed in sheep, cows, pigs, and humans and is encoded by the CLEC7A gene [75]. This lectin has previously been shown to stimulate both protective and non-protective immune responses [76,77]. In vitro studies of the dectin-1 interaction with *F. hepatica* have demonstrated that macrophages stimulated by *F. hepatica* excretory/secretory (FhES) products have up-regulated expression of PD-L2 resulting in T-cell anergy [78]. A non-protective immune response is stimulated, preventing the expulsion of the parasite, possibly through the extracellular signal-regulated kinase (ERK) and IL-10 signaling pathways. Phosphorylation of ERK is thought to be caused by the dectin-1 interaction with FhES products and spleen tyrosine kinase (Syk) [74], although when bone marrow-derived cells (BMDCs) from BALB/c mice were stimulated with adult *F. hepatica* total lysate (FhWWE) in the presence of the dectin-1 inhibitor laminarin, it did not prevent the binding or uptake of the parasite component by BMDCs [16]. In these studies, the specific glycoconjugate ligand remained unidentified. It is possible that the ligand was absent or present at much lower levels in the total lysate compared to FhES products, as no binding was observed between recombinant dectin-1-Fc and *F. hepatica* total lysate immobilized on a plate [53]. To clarify the cause of the differing responses between these *F. hepatica* preparations, the dectin-1-binding ligands must be identified and their differential expression across the extracts assessed. Overall, dectin-1 is thought to participate in Th2 stimulation but is not the only receptor playing a role in *F. hepatica* glycan interactions.

### 5.2. Macrophage Galactose-Type C-Type Lectin (MGL)

Macrophage galactose-type C-type lectin (MGL) is a type II transmembrane C-type lectin, also referred to as CLEC10A and CD301 [79]. One ortholog is present in humans (hMGL), whereas two are present in mice (mMGL1 and mMGL2). They are present on professional antigen-presenting cells such as dendritic cells and macrophages. Each of the mouse orthologs have different glycan specificities, although hMGL and mMGL2 both recognize GalNAc moieties. GalNAc is commonly expressed on helminths in glycan motifs such as the Tn antigen (αGalNAc O-Ser/Thr) or LacDiNAc (GalNAcβ1-4GlcNAc), which are both known ligands of MGL [53]. hMGL has been shown to interact with *S. mansoni* soluble egg antigen [80] and *Trichuris suis* glycopeptides [81]. Similarly, mMGL1 interacts with the cestode *T. crassiceps* and shows a role in the development of a protective Th1 immune response in mice [82]. Currently, it appears that each of the orthologs of MGL induces different adaptive immune responses by triggering different signaling pathways [79], as hMGL and mMGL2 appear to play a role in the modulation of a Th2 immune response.

One main study has been conducted to observe the in vitro effects of *F. hepatica* whole worm extract on hMGL-positive dendritic cells, as well as mMGL2+CD11C+F4/8010 mouse cells [53]. Firstly, it showed that hMGL present on human monocyte-derived dendritic cells in vitro binds to *F. hepatica* total lysate immobilized on a plate but not the soluble extract alone. Only when the dendritic cells had first been stimulated by TLR ligands (Pam3CSK4 and LPS) was an increase in IL-10, IL-6, and TNFα noted. This effect could be abrogated by pre-incubation with an anti-hMGL antibody, suggesting crosstalk between hMGL and TLR on dendritic cells. Additionally, if naïve CD4+ T cells were co-cultured with the dendritic cells, LPS and FhWWE were described as also showing Th2 polarization due to decreased IFNϒ production. Using a generic plant lectin VVL (GalNAc-specific lectin) and GalNAcase, it was demonstrated that this interaction was likely occurring with glycoconjugates containing terminal GalNAc [53]. More specifically, binding could be inhibited using anti-Tn-antigen antibodies [53], suggesting that hMGL interacts with Tn antigen present on FhWWE and causes TLR-induced maturation of dendritic cells.

In the same study, it was demonstrated that mMGL2-Fc, but not mMGL1-Fc, showed binding to FhWWE and that the binding of mMGL2 showed a similar pattern to that of hMGL [53]. Additionally, when BALB/c mice were experimentally infected with 10 metacercariae, mMGL2 CD11c+ F4/8010 cells were recruited to the peritoneum and were shown to up-regulate IL-10, TNFα, and TGFβ. Similar to the anergy effects caused by hMGL, mMGL2 also had the ability to cause a decrease of IFNϒ secreted by CD4+ T cells. This is a Th2-type immune response, although the exact mechanism is unknown.

Although the sheep MGL proteins have not yet been characterized, sheep do possess a CLEC10A gene that has been shown to be up-regulated in liver tissue of infected sheep at 8 weeks post-infection compared to the control group [83]. It would be very interesting to study this protein further in this economically significant host of *F. hepatica* to determine if multiple orthologs exist, if it has similar ligands to hMGL, and if this interaction results in similar effects to those seen in other species.

### 5.3. Mannose Receptor

Mannose receptor (MR) is a type I membrane protein (also referred to as CD206) that has been identified on macrophages and dendritic cells from mice and humans. It differs from the previously mentioned C-type lectins in that it has more than one binding site. It has a short cytoplasmic domain that is involved in receptor internalization via clathrin-coated vesicle formation. The extracellular portion of the receptor is made up of three separate domains, and at the N-terminus, there is a cysteine-rich domain involved in binding to sulphated sugars that is independent of Ca^2+^. Secondly, there is a fibronectin type II domain involved in collagen binding and, lastly, there are 8–10 C-type lectin-like domains, only some of which bind to terminal mannose, fucose, and GlcNAc [84]. MRs on peritoneal macrophages of mice were first shown to interact with *F. hepatica* ES in vitro by Guasconi, et al. [17] by using an anti-MR antibody, which demonstrated a partial reduction in IL-10 and TGF-β levels produced by macrophages compared to levels released due to FhES products alone. Additionally, in vivo studies of mice intraperitoneally injected with mannan and laminarin, inhibitors of MR and dectin-1, respectively, showed a partial reduction of the elevated IL-10 and TGF-β levels before oral infection of metacercariae [17].

In another study using BMDCs from MR knockout mice, it was demonstrated that MR is involved in the binding of FhTeg (a complex detergent extract of the *F. hepatica* tegument that also contains ES molecules) to the surface of dendritic cells; however, this did not have an effect on the expression of SOCS3 or LPS-induced IL-12p70 [36]. The role played by MR in the binding of FhTeg was also demonstrated in MR transfected CHO cells. Binding could be inhibited by pre-incubation with EGTA, mannan, and sulphated GalNAc [36], which is interesting as sulphated glycans had been reported in *F. hepatica* from the early anionic staining of the *F. hepatica* glycocalyx but could not be identified by mass spectrometry [36,42]. Aldridge and O’Neill [85] have suggested that MR facilitates the communication between dendritic cells and CD4+ T cells that is not mediated by cross-presentation but instead by direct cell-to-cell communication between the MR of dendritic cells and CD45 on CD4+ T cells. Similarly, using MR knockout mice, a decrease in the expression of negative regulating transcription factors was also shown [85]. These preliminary studies suggest that MR in combination with other C-type lectins is playing a role in the interaction between the host and the *F. hepatica* tegument and ES.

### 5.4. Dendritic Cell-Specific ICAM-3 Grabbing Non-Integrin (DC-SIGN)

Dendritic cell-specific ICAM-3 grabbing non-integrin (DC-SIGN), also referred to as CD209, is a type II membrane C-type lectin. As the name implies, it is expressed on dendritic cells. It is involved in the surveillance of ‘self’ and foreign antigens as well as triggering the adaptive immune response via affecting the regulation and differentiation of T cells [86]. Following interaction between pathogens and DC-SIGN, many effects on dendritic cells have been demonstrated, such as internalization, antigen presentation, as well as TLR-induced maturation of dendritic cells [18]. Active recombinant DC-SIGN has been passed over a commercial glycan array, and it was demonstrated that DC-SIGN has the greatest affinity for multivalent Lewis X (Galβ1,4(Fucα1,3)GlcNAc-R) (Le^x^) glycans but also binds with moderate affinity to oligomannose, diantennary Le^x^, and LacdiNAc-Fuc structures [86]. Recognition of oligomannose glycans was confirmed by DC-SIGN recognition of oligomannose surface-expressed CHO cells. By using both transfected CHO cells and an array format, a complete lack of binding to core fucosylated glycans was demonstrated [86].

DC-SIGN has also been demonstrated to play a role in the innate defense against *S. mansoni*, *Leishmania* spp., and *T. suis* infection [81,87,88]. Recently, human monocyte-derived dendritic cells activated with LPS and stimulated with FhTeg demonstrated an increased production of TLR-induced IL-10 and IL-27p28, two interleukins involved in the induction of T-cell anergy and regulatory T cells. Similarly, when CD4+ T cells were co-cultured with human monocytes expressing DC-SIGN, they showed a reduction in the excretion of IFNϒ, similar to that observed in the hMGL studies [18]. Using imaging flow cytometry, both MR and DC-SIGN were demonstrated to play a part in the binding and uptake of FhTeg by human monocyte dendritic cells. However, when cells were first incubated with anti-C-type lectin antibodies (MR, DC-SIGN, and MGL), only incubation with anti-DC-SIGN showed any ability to restore the levels of IL-10 and IL-27p28 back to that of the control. Additionally, this incubation also partially restored the production of IFNϒ by CD4+ T cells. These effects were reduced by the addition of EGTA, which inhibits sugars and glycosidases such as mannosidase and fucosidase, illustrating that it was in fact a C-type lectin recognizing mannose and fucose involved in these events [18]. In another study using *F. hepatica* whole worm extract, DC-SIGN was shown to mediate IL-27-dependent T-cell differentiation into follicular T-helper cells [89]. This was thought to be triggered by terminal fucose glycans such as LDNF (fucosylated LacdiNAc). However, it is now known that *F. hepatica* do not appear to possess the glycosyltransferase genes involved in the addition of terminal fucose; additionally, mass spectrometry studies suggest only modifications of the core fucose are present in *F. hepatica* [18,28,36,37]. Although it had been speculated that terminal fucose could be present on O-glycans or glycolipids, this is yet to be demonstrated in *F. hepatica*. It would appear instead that DC-SIGN is interacting with the oligomannose structures of *F. hepatica* and playing a part in polarizing host immunity to a Th2/T-helper cell immune response.

### 5.5. Galectin

Another family of lectins is the β-galactoside binding lectins commonly known as galectins. They are secreted via the non-classical pathway by both the host and helminth parasites and are believed to play an important role in pathogen surveillance and innate immunity [90,91]. Of the 15 known galectin types, galectin-1, -3, -9, -11, and -14 derived from host species have been shown to play a role in the interaction with parasites [92]. Galectin-11 and galectin-14 are both produced by ruminants and contain a single carbohydrate recognition domain [93,94]. Both galectin-11 and -14 have been identified in the bile and bile duct tissue of naturally infected sheep [95].

Galectin-14 is constitutively expressed in eosinophils and is secreted following a stimulus, whereas galectin-11 is specifically up-regulated following infection. More specifically, galectin-11 is secreted from epithelial cells of the gastrointestinal tract following nematode infection or epithelial cells of the bile ducts of *F. hepatica*-infected sheep [93,95]. Additionally, through transcriptional studies, galectin-14 has been shown to be up-regulated in peripheral mononuclear bone marrow cells at 2 and 8 weeks post-infection and in the liver tissue of infected animals [83,96]. Although native galectin-14 has not been found attached to the surface of *F. hepatica*, likely due to the nature of their tegument turnover, galectin-14 has been shown to bind to the surface of cryosections of adult *F. hepatica* in a carbohydrate-dependent manner, as well as being able to associate with FhES products [5]. Similarly, when galectin-14 was immobilized to a sepharose resin and adult fluke lysate passed over the resin, galectin-14 was found to interact with a large number of proteins, many of those being membrane proteins, including some previously described vaccine candidates [97]. Interestingly, the most abundant protein identified was an uncharacterized C-type lectin that has a Ser/Thr-rich C-terminus, supporting the hypothesis that galectin-14 may be interacting with mucin-type glycoproteins [97,98]. Surprisingly, when this experiment was performed in parallel with recombinant galectin-11, far fewer proteins were captured [97]. To date, a correlation between *F. hepatica* viability in vivo and galectin-11 has not been revealed, although it has been identified in the bile of experimentally infected animals [98]. Due to the lack of convincing natural resistance in any species to *F. hepatica* and the recruitment of eosinophils to the site of infection, it has been speculated that flukes could be manipulating host galectin-14 and eosinophils for their survival [92]. This warrants further investigation.

### 5.6. Serpin

It has been known for some time that *F. hepatica* extracts can inhibit the complement pathways of the host. The exact mechanism has not been fully elucidated, although sloughing of the tegument has been shown to shed antigen/antibody complexes, and the tegument display of CD59 orthologs is believed to inhibit the formation of membrane attack complexes [99]. However, considering the tegument of *F. hepatica* NEJ and adult life stages is dominated by oligomannose- and paucimannose-type N-glycans, it is surprising that the lectin complement pathway is not strongly activated. It is believed that *F. hepatica* employs a mechanism to block host mannose/mannan binding lectins (MBLs) binding to their surface [100]. Additionally, serpin 1 and 2 released by the fluke have been demonstrated to inhibit host (human) MBL-associated serine proteases (MASPs), preventing further complement deposition [100]. Together, these findings suggest that *F. hepatica* has evolved multiple strategies to evade complement-mediated destruction, effectively suppressing both classical and lectin pathway activation despite the presence of glycans typically recognized by host immune lectins.

## 6. Future Directions

The exact role specific parasitic glycans play is not fully understood, although they are believed to stimulate both the innate and adaptive immune response. In this review, a number of glycan–protein interactions have been demonstrated that are thought to play a role in stimulating an anti-inflammatory immune response and facilitating immune evasion. Better characterization of the receptors and glycan ligands involved in these responses could potentially be harnessed in the future to treat inflammatory immune disorders [101]. Altered glycosylation patterns can act as an immune evasion mechanism, the question therefore remains if *F. hepatica* alters its surface-exposed glycans depending on the definitive or intermediate host.

Many glycan types have not yet been explored in *F. hepatica,* including glycosaminoglycans, O-mannosylation, C-mannosylation, and O-GlcNac, each with the potential to reveal parasite-specific glycan structures. In other helminth infections, specific anti-glycan antibody responses have been documented across multiple host species, including humans, mice, and monkeys infected with *S. mansoni* [102,103]; rats and monkeys infected with *S. japonicum* [104,105]; and in infections with various filarial nematode species [106]. To date, an anti-glycan antibody response against *F. hepatica* glycans has not been thoroughly demonstrated. Following more in-depth characterization of *F. hepatica* glycans, this knowledge of antigenic glycans could help in the development of glycoengineered vaccines that could potentially aid stability, antigen presentation, and antigenicity of the vaccine candidate [20]. Previous vaccine candidates that are now known to be glycosylated could potentially be expressed in alternative expression systems that allow for paucimannose- and oligomannose-type glycosylation (the most abundant N-glycans detected in *F. hepatica* to date); the products of these different glycosylation systems and their deglycosylated counterparts could be compared in a vaccine trial.

Ideally, our knowledge of *F. hepatica* glycan compositions and structures first needs to be extended not only to the dominant glycans but also to the less abundant ones. This review has demonstrated that previous lectin staining has suggested that more complex glycans are present, and only some of these types of structures have been identified with mass spectrometry. In order to better characterize the low-abundance glycans, better strategies are required to minimize signal suppression by highly abundant glycans and to achieve effective isomeric and isobaric separation. A more detailed knowledge of the different isomer present will shed light on the various glycosylation pathways implemented by the parasite, providing more defined targets. The most recent glycan library created from NEJ extracts has allowed glycopeptide analysis to be conducted, shedding light on the heterogeneity of the different glycoforms of glycosylated vaccine candidates [50]. For example, many of the cathepsins are glycosylated, predominantly with paucimannose- and oligomannose-type N-glycans, which can also be modified with one or more PC residues [50]. Glycoengineering these glycoproteins in novel expression systems to display the same glycans as the native glycoprotein, followed by evaluation in vaccine trials, could result in greater efficacy than previous recombinant antigens.

Previous work has only begun to explore the spatial distribution of glycolipids in immature and adult flukes within the rat host using mass spectrometry imaging [64]. However, multi-omic imaging approaches, including N-glycan imaging (achievable due to the lack of α1-3 core fucose), could offer greater spatial context to N-glycan distribution than the current comparison between tegument and somatic extracts. This could enable the confirmation and/or identification of novel surfaced-exposed antigens. This area is still in its infancy, and there is much still to be discovered and understood, not only for *F. hepatica* but also for other parasites of importance.

## 7. Conclusions

It appears that host lectins are each stimulating a similar immune response characterized by the heightened expression of IL-10 by antigen-presenting cells and diminished IFN-γ expression by T cells. Due to the long history of co-evolution, it seems that *F. hepatica* has utilized these host immune lectins in combinations to help stimulate a coordinated regulator/Th2 immune response, although there are many other contributing factors involved [12]. With a deeper comprehension of the glycans of *F. hepatica* and their interactions with the innate and adaptive immune response, we can adopt a more holistic approach to designing vaccine candidates and drug targets that take into consideration the glycosylation of the native protein target. With the plethora of recombinant expression systems now available for glycoengineering glycoproteins, understanding native glycosylation and its specific interaction with the immune system is poised to enhance the likelihood of vaccine success.

## Data Availability

No new data were created or analyzed in this study. Data sharing is not applicable to this article.

## Abbreviations

The following abbreviations are used in this manuscript:

2-AA	2-aminobenzoic acid
AAL	*Aleuria aurantia* lectin
ALG	Asparagine-linked glycosylation
BaSII	*Biomphalaria alexandrina* lectin two
BMDCs	Bone marrow derived cells
CHO	Chinese hamster ovary
CID	Collision-induced dissociation
CMP	Cytidine monophosphate
ConA	Concanavalin A
CPA	Chickpea lectin
DBA	*Dolichos biflorus* agglutinin
DC-SIGN	Dendritic cell-specific ICAM-3 grabbing non-integrin
Dol-P	Dolichol phosphate
DSA	*Datura stramonium* agglutinin
ECD	Electron capture dissociation
ECL	*Erythrina cristagalli* lectin
EDT	Electron transfer dissociation
ELISA	Enzyme-linked immunosorbent assay
ER	Endoplasmic reticulum
ERK	Extracellular signal-regulated kinase
ES	Excretory/secretory
ESI-MS	Electrospray ionization mass spectrometry
EVs	Extracellular vesicles
FhCatB1	Cathepsin B1
FhCatL3	Cathepsin L3
FhES	*Fasciola hepatica* excretory/secretory
FhTeg	*Fasciola hepatica* tegument extract
FhWWE	*Fasciola hepatica* whole worm extract
Fuc	Fucose
GAG	Glycosaminoglycan
GalNAc	N-acetylgalactosamine
GDP-Fuc	Guanosine diphosphate fucose
GDP-Man	Guanosine diphosphate mannose
Glc	Glucose
GlcA	Glucuronic acid
GlcNAc	N-acetylglucosamines
GNL	*Galanthus nivalis* lectin
GPI	Glycosylphosphatidylinositol
GSL-1-B4	*Griffonia simplicifolia* lectin one isolectin B4
GSL-I	*Griffonia simplicifolia* lectin one
GSL-II	*Griffonia simplicifolia* lectin two
GTP	Guanosine triphosphate
Hex	Hexose
HexNAc	N-acetylhexosamine
H-Gal-GP	*Haemonchus*-galactose-containing glycoprotein
HHA	Hippeastrum hybrid (amaryllis) lectin
IFN_ϒ_	Interferon gamma
IL	Interleukin
LacDiNAc	GalNAcβ1-4GlcNAc
LacNAc	N-acetyllactosamine
LAP	Leucine aminopeptidase
LCA	*Lens culinaris* agglutinin
LC-MS/MS	Liquid Chromatography–tandem mass spectrometry
LDNF	Fucosylated LacdiNAc (GalNAcβ1,4(Fucα1,3)GlcNAc-R)
LEL	*Lycopersicon esculentum* lectin
Le^x^	Lewis X (Galβ1,4(Fucα1,3)GlcNAc-R)
LGALS-11	Galectin-11
LGALS-14	Galectin-14
LNnT	Lacto-N-neotetrasose
LPS	Lipopolysaccharides
LTA	*Lotus tetragonolobus* lectin
MALDI-FT-ICR MS	Matrix-assisted laser desorption/ionization Fourier transform ion cyclotron resonance mass spectrometry
MALDI-TOF MS	Matrix-assisted laser desorption ionization–time of flight mass spectrometry
MALDI-TOF MS/MS	Matrix-assisted laser desorption ionization–time of flight tandem mass spectrometry
MAL-I	*Maackia amurensis* lectin one
MAL-II	*Maackia amurensis* lectin two
Man	Mannose
MHC	Major histocompatibility complex
MOA	*Marasmium oreades* agglutinin
MPA	*Maclura pomifera* agglutinin
MR	Mannose receptor
MS	Mass spectrometry
MS/MS	Tandem mass spectrometry
NEJ	Newly excysted juvenile
Neu5Ac	N-acetylneuraminic acid
Neu5Gc	N-glycolylneuraminic acid
NMR	Nuclear magnetic resonance
NPA	*Narcissus pseudonarcissus* lectin
OST	Oligosaccharyltransferase
PBMCs	Peripheral blood mononuclear cells
PC	Phosphorylcholine
PD-L2	Programmed cell death ligand 2
PHA-E	*Phaseolus vulgaris* agglutinin-E
PHA-L	*Phaseolus vulgaris* agglutinin-L
PNA	Peanut agglutinin
PNGase A	Peptide:N-glycosidase A
PNGase F	Peptide:N-glycosidase F
PRR	Pattern recognition receptors
PSA	*Pisum sativum* agglutinin
RCA-I	*Ricinus communis* agglutinin
SBA	Soybean agglutinin
SJA	*Sophora japonica* agglutinin
SNA-I	*Sambucus nigra* lectin one
SNA-II	*Sambucus nigra* lectin two
STL	*Solanum tuberosum* lectin
s-WGA	Succinyl Wheat germ agglutinin
Syk	Spleen tyrosine kinase
TCBZ	Triclabendazole
TGF-β	Transforming growth factor β
TLR	Toll-like receptor
Tn antigen	GalNAc Ser/Thr
TNFα	Tumor necrosis factor alpha
UDP	Uridine diphosphate
UEA-1	*Ulex europaeus* agglutinin one
VRA	*Vigna radiata* Lectin
VVL	*Vicia villosa* lectin
WFA	*Wisteria floribunda* agglutinin
WGA	Wheat germ agglutinin
